# Kinase-Inhibitors in Iodine-Refractory Differentiated Thyroid Cancer—Focus on Occurrence, Mechanisms, and Management of Treatment-Related Hypertension

**DOI:** 10.3390/ijms222212217

**Published:** 2021-11-12

**Authors:** Anne Christine Kaae, Michael C. Kreissl, Marcus Krüger, Manfred Infanger, Daniela Grimm, Markus Wehland

**Affiliations:** 1 Department of Biomedicine, Aarhus University, Ole Worms Allé 4, 8000 Aarhus, Denmark; anne.christine.kaae@gmail.com (A.C.K.); daniela.grimm@med.ovgu.de (D.G.); 2 Division of Nuclear Medicine, Department of Radiology and Nuclear Medicine, Otto von Guericke University, Leipziger Str. 44, 39120 Magdeburg, Germany; michael.kreissl@med.ovgu.de; 3 Department of Microgravity and Translational Regenerative Medicine, Otto von Guericke University, Universitätsplatz 2, 39106 Magdeburg, Germany; marcus.krueger@med.ovgu.de (M.K.); manfred.infanger@med.ovgu.de (M.I.); 4 Clinic for Plastic, Aesthetic and Hand Surgery, Otto von Guericke University, Leipziger Str. 44, 39120 Magdeburg, Germany

**Keywords:** thyroid cancer, multikinase inhibitors, sorafenib, cabozantinib, lenvatinib, adverse effects, hypertension

## Abstract

Differentiated thyroid cancer (DTC) usually has a good prognosis when treated conventionally with thyroidectomy, radioactive iodine (RAI) and thyroid-stimulating hormone suppression, but some tumors develop a resistance to RAI therapy, requiring alternative treatments. Sorafenib, lenvatinib and cabozantinib are multikinase inhibitors (MKIs) approved for the treatment of RAI-refractory DTC. The drugs have been shown to improve progression-free survival (PFS) and overall survival (OS) via the inhibition of different receptor tyrosine kinases (RTKs) that are involved in tumorigenesis and angiogenesis. Both sorafenib and lenvatinib have been approved irrespective of the line of therapy for the treatment of RAI-refractory DTC, whereas cabozantinib has only been approved as a second-line treatment. Adverse effects (AEs) such as hypertension are often seen with MKI treatment, but are generally well manageable. In this review, current clinical studies will be discussed, and the toxicity and safety of sorafenib, lenvatinib and cabozantinib treatment will be evaluated, with a focus on AE hypertension and its treatment options. In short, treatment-emergent hypertension (TE-HTN) occurs with all three drugs, but is usually well manageable and leads only to a few dose modifications or even discontinuations. This is emphasized by the fact that lenvatinib is widely considered the first-line drug of choice, despite its higher rate of TE-HTN.

## 1. Introduction

Thyroid cancer (TC) comprises a malignant tumor in the thyroid gland. It is the most common cancer in the endocrine system, with approximately 585,000 new cases in 2020 [1], thus accounting for 3% of all cancers. In about 3/4 of the cases women are affected, with an incidence rate of 3.1 per 100,000 for men and 10.1 for females per year. The incidence rate of TC is increasing, with one of the reasons probably being more advanced technology and better diagnostic tools. Interestingly, in the US, mortality rates for differentiated TC between 1994 and 2013 have also seen an increase, i.e., in papillary TC by 2.9% per year [2], which implies that there is a need for improvement in the treatment options for this disease. The subtypes of TC are differentiated thyroid cancer (DTC), medullary thyroid cancer (MTC), anaplastic thyroid cancer (ATC) and poorly differentiated thyroid cancer (PDTC) [3,4]. DTC accounts for 95% of TC cases [5] and can be further subcategorized into follicular thyroid cancer (FTC), papillary thyroid cancer (PTC) and Hürthle cell thyroid cancer (HTC), of which PTCs are the most common. DTC originates in the follicular cells of the thyroid [5]. Under physiological conditions, these cells line the colloid follicles, concentrate iodine, and produce thyroid hormones [4,6].

Oncogenic pathways, important for different aspects of tumorigenesis, are involved in the development of TC. Some of the intracellular signaling pathways involved are RAS/RAF/MAPK and PI3K/AKT/mTOR. These factors are important regulators of cell function and can activate, among other ligands, various receptor tyrosine kinases (RTKs), which has effects on progression, proliferation and tumorigenesis [7,8]. Some of the involved RTKs are epidermal growth factor receptors (EGFR), platelet-derived growth factor receptors (PDGFR), hepatocyte growth factor receptor (HGFR or MET) and vascular endothelial growth factor receptor (VEGFR). PTC mutations such as BRAF (50–70%), RET (30%) and RAS (10%) are the most common and are associated with a poor prognosis, increasing recurrence and radioactive iodine (RAI) refraction [5,9,10,11]. Mutations at any level of the pathways can lead to constitutive activation, overexpression, or other oncogenic effects [8,12]. Treatment strategies of TC rely on both anti-proliferative and anti-angiogenic properties, as tyrosine kinases (TKs) are important in these two areas of the TC development of factors involved in proliferation, survival, cell cycle, and intracellular growth pathways, as well as angiogenic factors, i.e., those leading to neo-angiogenesis [13,14,15]. However, although first breakthroughs in the form of marketable drugs have been achieved, the field of developing TKI against refractory DTC is still evolving [13,16].

Proteins of the vascular endothelial growth factor (VEGF) family stimulate the formation of new blood vessels (angiogenesis), and the proliferation of endothelial cells, etc., via binding to the VEGFR, which is necessary for the growth, development, and metastasis of tumors. The most important member of the VEGFR family associated with tumor angiogenesis is VEGFR-2 [17,18,19,20]. Under normal circumstances, VEGFR-2 expression is low, but it can be upregulated during pathophysiological tumor angiogenesis. VEGF and VEGFR expressions are not restricted to endothelial cells, as a large number of cells express VEGF and its receptors [21,22,23,24]. Tumor cells secrete VEGF and VEGFR, and the VEGF signaling in tumor cells is both auto- and paracrine. Increased levels of VEGF are negatively correlated with overall survival (OS) and progression-free survival (PFS) [23,25]. Furthermore, the presence of VEGF-A and VEGFr-2 polymorphisms can be a predictor for the therapy response and recurrence of DTC [26,27].

c-MET is an RTK that binds the ligand hepatocyte growth factor (HGF), and RET is a protooncogene that encodes the RET receptor. The activation of these receptors will induce a range of intracellular signaling pathways, including the pathways involved in proliferation, migration, and invasion [28,29].

The primary treatment for DTCs is surgical, i.e., total thyroidectomy in order to remove the tumor. TSH suppression may help to reduce the risk of tumor growth, but this can sometimes lead to tachycardia and possibly to slightly increased blood pressure (BP). RAI is regularly used as an adjuvant treatment, but also can be applied as a highly specific and effective tumor-targeted therapy. Accordingly, the ability to concentrate iodine is critical for the survival rates of TC patients. The prognosis for patients diagnosed with DTC is usually very good, with a 5-year survival rate of more than 90%, and even a 50% survival rate when distant metastases occur or are present initially [28,30,31,32].

Unfortunately, some tumor cells become or are initially refractory to RAI treatment due to a loss/lack of function of the Na/I symporter (NIS) and an upregulation of tyrosine kinase receptors, among other things [6,12]. RAI refractory DTC (RR- DTC) is defined by a loss or deficit of the ability to take up RAI, occurring either initially in metastases, as the absence of RAI uptake after treatment with RAI, or as disease progression despite RAI uptake. The absence of RAI uptake can occur in only some tumor manifestations or in all. The prognosis for RR-DTC is poor, with a 5-year survival rate of 66% and a 10-year survival rate of only 10%. RAI refractoriness is seen in about one-third of DTC patients with recurrence or metastases [9,30,33,34,35].

### Multikinase Inhibitors

Even though RR-DTCs account for only a few new cancer cases each year [35], it is extremely important to find an efficient treatment for these cancers, due to their aggressive nature and poor prognosis. A better understanding of the progression and tumorigenesis have led to the development of specific targeted therapies, e.g., multikinase inhibitors (MKIs) and tyrosine kinases inhibitors (TKIs) [31,36,37,38].

MKIs target different signaling pathways and oncogenes, which are affected by the TC and involved in proliferation, survival, motility, migration, invasion, etc. RTKs are enzymes that phosphorylate proteins important in intracellular signaling pathways [31,39], and the inhibition of these tyrosine kinases associated with cellular receptors leads to the inhibition of tumor growth [13,40].

So far, three drugs have been approved for the treatment of RR-DTC: sorafenib, lenvatinib and cabozantinib (Table 1).

**Figure 1:**
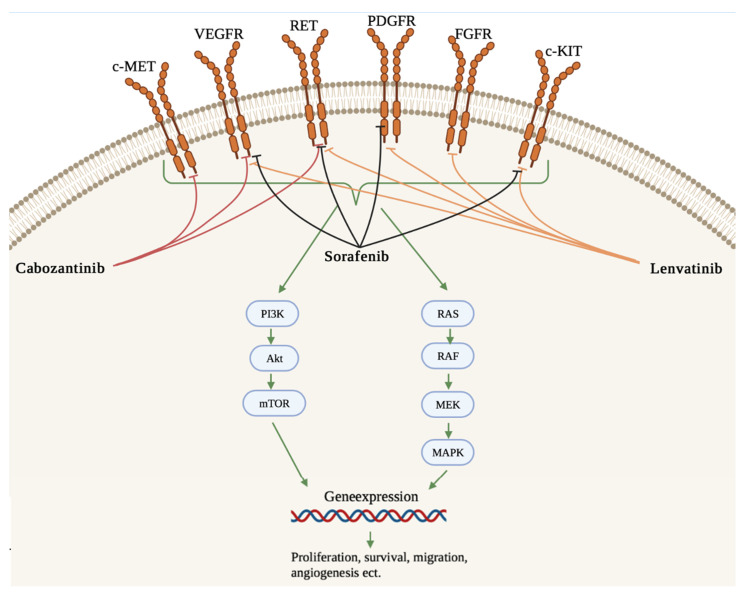
Targets of sorafenib, lenvatinib and cabozantinib. The intracellular signaling pathway MAPK and the PI3K/AKT/mTOR pathway are involved in the development of TC. VEGFR (vascular endothelial growth factor receptor), PDGFR (platelet-derived growth factor receptor), FGFR (fibroblast growth factor receptor), RET (rearranged during transfection), c-KIT (stem cell factor receptor), RAS (rat sarcoma protein), MET (Hepatocyte growth factor receptor), RAF (rapidly accelerated fibrosarcoma kinase), MEK (mitogen-activated protein kinase kinase), MAPK (mitogen-activated protein kinase), PI3K (phosphoinositide 3-kinase), AKT (protein kinase B), mTOR (mammalian target of rapamycin) [9,18,45]. Adapted from [9,18,49]. The figure was drawn using Biorender.com (accessed on 21 October 2021).

Lenvatinib is an oral TKI that targets receptors and oncogenes, e.g., VEGFR1-3, PDGFRα, RET, c-KIT, FGFR1-4 [32,35,48]. Cabozantinib is an oral MKI targeting VEGFR-2, c-MET and RET [31], and sorafenib targets VEGFR-2 and -3, PDGFR, c-Kit, RET/PTC and RAF [41] (Figure 1).

Sorafenib (NEXAVAR^®^) interacts with the intracellular (CRAF, BRAF and mutant BRAF) and cell surface kinases cKIT, FLT-3, VEGFR-2, VEGFR-3, and PDGFR-β, as well as Raf kinase [18,25]. It is a small molecule inhibitor. Sorafenib simultaneously targets the Raf/Mek/Erk pathway.

Moreover, these kinases are involved in angiogenesis. Therefore, sorafenib can reduce the blood supply to the tumor. In addition, genetic transcription involving cell proliferation and angiogenesis is inhibited [50]. Sorafenib inhibits tumor growth and angiogenesis in models of thyroid cancer [51].

The MKI lenvatinib (Lenvima^®^) inhibits the fibroblast growth factor receptors (FGFR) 1, 2, 3 and 4, the three main vascular endothelial growth factor receptors VEGFR1, 2 and 3, platelet-derived growth factor receptor (PDGFR) alpha, and finally c-Kit, as well as the RET proto-oncogene. Thus, lenvatinib inhibits angiogenesis and reduces cancer growth [52]. These receptor tyrosine kinases are located in the cell membrane and are involved in the signal transduction of various biological processes, such as differentiation, migration, cell proliferation and apoptosis, as well as pathogenic angiogenesis, lymphogenesis, cancer growth and metastasis.

Cabozantinib (Cabometyx^®^) inhibits specific receptor tyrosine kinases, such as VEGFR-1, -2 and -3, KIT, TRKB, FLT-3, AXL, RET, MET, and TIE-2. This action results in the suppression of the biological processes angiogenesis, tumorigenesis, cancer progression and metastasis. Cabozantinib is a potent inhibitor of RET and effectively inhibits the growth of an MTC tumor cell model in vitro and in vivo [53].

All three drugs showed significant improvements in PFS compared to a placebo in their respective pivotal phase 3 trials (Table 2).

## 2. Methods

The literature search was performed using the PRISMA guidelines (Preferred Reporting Items for Systematic Reviews and Meta-Analyses) [64] and by using the PubMed database [65]. The PubMed database search was conducted on 10 May 2021 and on 17 October 2021.

The searches for clinical trials regarding lenvatinib and cabozantinib were conducted in the clinicaltrials.gov database. The searches were conducted on 05 May 2021 for lenvatinib studies, on 11 May 2021 for cabozantinib, and on 17 October 2021 for sorafenib by using the clinicaltrial.gov database.

### 2.1. Eligibility Criteria

The studies included in this review regard the safety and/or the efficacy of MKI treatment of RR-DTC in patients above 18 years of age. The exclusion criteria were articles published before 1 January 2016, languages other than English, and studies in vitro or on animals. Furthermore, meta-analyses, case reports and systematic reviews were not included.

The eligibility criteria for the clinical trials were trials studying the treatment of patients with RR-DTC. The exclusion criteria were trials conducted on cell cultures or animals, results published or last updated before 1 January 2016, thyroid cancers other than RR-DTC, and focuses other than the safety, efficacy, or adverse effects (AE) of hypertension.

### 2.2. Search

Literature searches in the PubMed database were conducted using the search terms: (“thyroid neoplasms”[Mesh] OR “thyroid carcinoma”[Mesh] OR “thyroid cancer, papillary”[Mesh] OR “thyroid cancer, Hürthle cell” [Mesh] OR “thyroid cancer, follicular” [Mesh] OR “thyroid cancer, differentiated” [Mesh] OR “thyroid cancer”[title/abstract]) AND (“multikinase inhibitor” OR “tyrosine kinase inhibitor” OR “lenvatinib” OR “cabozantinib” OR “sorafenib” OR “MKI/TKI” OR “lenvatinib/cabozantinib/sorafenib”)

Searches in the clinicaltrials.gov database [66] employed “thyroid cancer” as terms for conditions or diseases combined with “lenvatinib”, “cabozantinib” or “sorafenib” as other terms, respectively.

### 2.3. Study Selection

The literature search resulted in 973 articles. A total of 958 articles were excluded according to the eligibility criteria by screening the title, abstract and publication date; 15 articles met the inclusion criteria.

## 3. Results

### 3.1. Multikinase Inhibitor Treatment Causes Hypertension

**Figure 2:**
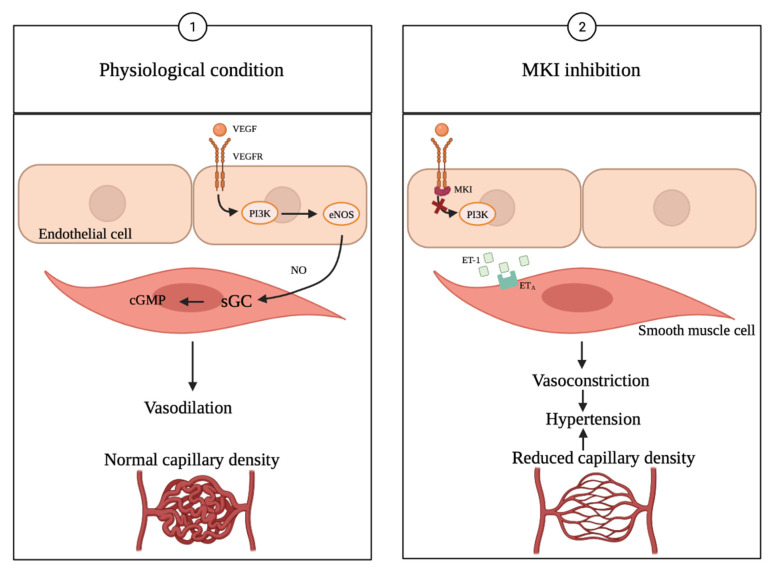
MKI treatment induced hypertension. Inhibition of VEFGR causes hypertension, as VEGFR receptors are present on both healthy vascular endothelial cells and in cancer cells, causing the MKI treatment to also affect healthy endothelial cells [18] (adapted from [18,75]). NO production is inhibited, and the endothelin-1 pathway is activated leading to vasoconstriction. VEGF is important for the integrity of the capillary network and inhibition of the VEGFR leads to reduction in capillary density [75]. VEGF (vascular endothelial growth factor), VEGFR (vascular endothelial growth factor receptor), PI3K (phosphatidylinositol-3-kinase), eNOS (endothelial nitric oxide), sGC (soluble guanylate cyclase), cGMP (cyclic guanosine monophosphate), ET-1 (endothelin-1), ET_A_ (endothelin receptor type A) [18,75]. The figure was drawn using Biorender.com.

The inhibition of VEGFRs is known to cause hypertension [3,17]. The mechanisms leading to the development of hypertension are not fully understood, but some suggestions as to the mechanisms have been accepted. One of the discussed mechanisms is that VEGFR inhibition induces the interruption of survival signaling in the endothelial cells. This can result in apoptosis and capillary rarefaction, leading to reduced angiogenesis and thereby to an increase in vascular resistance [17,67,68], which, according to Ohms law (blood pressure = cardiac output × total peripheral resistance), increases the BP (Figure 2).

Another possible mechanism could be that the inhibition of the VEGF leads to vasoconstriction because of the decreased activation of endothelial nitric oxide synthase inhibiting the production of nitric oxide (NO) and $PGI_{2}$ in the endothelial cells, and the simultaneous activation of the endothelin system [68,69,70], leading to a constriction of the smooth muscle cells. The endothelin-1 pathway’s (ET-1) activation also causes hypertension [17,71]. The hypothesis of the inhibition of NO production is supported by Sueta et al. [71], who measured decreased serum levels of NO compared with pretreatment levels. The function of the vascular endothelial cells is impaired during treatment with VEGFR inhibitors [71].

Furthermore, VEGFR also regulates endothelial cell function within renal glomeruli. VEGFR inhibition influences, e.g., glomerular filtration and salt and water retention, which can affect the BP and lead to hypertension [67,72].

The adrenergic system and the renin–angiotensin–aldosterone axis may also be involved in the development of treatment-emerged hypertension (TE-HTN), but different findings suggest that this is not the primary mechanism associated with hypertension caused by VEGFR inhibition [20,68,73,74].

### 3.2. Clinical Study Findings

One of the most common AEs when treating cancer with MKIs is arterial hypertension [17,18,25,31,76]. Treatment-related hypertension can be divided into different grades of AEs based on the different hypertensive stages correlating with the classifications of BP (grade 1 AE is defined as SBP 120–39 mmHg and DBP 80–89 mmHg, grade 2 AE as SBP 140–159 mmHg and DBP 90–99 mmHg, grade 3 as SBP >160 mmHg and DBP >100 mmHg, and grade 4 as life threatening hypertension (Table 3)) [17,77].

TEAE hypertension is usually manageable, either by dose reduction/interruption or by using antihypertensive drugs. However, antihypertensive drugs may interfere with MKI treatment, for example by inducing or blocking the liver enzymes involved in the degradation of MKI, or by synergistic effects on QTc prolongation with MKI. This is why further research on this topic is needed. In any case, it is important to be aware of the presence and severity of hypertension. Regular BP monitoring is required [74,79,80,81,82]. The most common AEs are shown in Table 4.

TE-HTN occurred in all three phase 3 studies with different frequencies. The COSMIC-311 trial registered a total of 28% of patients receiving cabozantinib with all-grade TE-HTN (9% ≥ grade 3) [54]. The DECISION trial reported a total of 50.3% of patients receiving sorafenib with all-grade TE-HTN (9.7% grade ≥ 3) [56], and the largest percentage was found in the SELECT trial [58] with 67.8% of all patients receiving lenvatinib experiencing all-grade TE-HTN (41.8% grade ≥ 3) (Table 4). In a later analysis of the same cohort, slightly different numbers were reported (73% all-grade TH-HTN, 44% grade ≥ 3) [17].

In the COSMIC-311 trial, TE-HTN was not among the top three causes for dose reduction (<7%), and only one patient discontinued treatment due to hypertension. The median time to the first dose reduction was 57 days (interquartile range 35–90 days), indicating that hypertension and other AEs occurred early during the study, was subsequently manageable, and did not interfere severely with the treatment regimen [54].

Of the 207 patients in the sorafenib DECISION trial, 16 (8.7%) needed a treatment interruption, 12 (5.8%) underwent dose reduction, and 1 patient (0.5%) discontinued treatment due to TE-HTN. The highest incidence of TE-HTN occurred in treatment cycles 1 and 2 with about 22% and 9%, respectively. In cycles 3–9, the incidence was <5%. The prevalence of TE-HTN remained essentially constant, with fluctuations between 22% and 25%. Similar tendencies were observed for the severity of TE-HTN, while percentages of grades 1 and 2 varied more strongly over time, and the prevalence of grade 3 hypertension remained consistently within 2–5% over the course of the whole study [55]. This indicates that a combination of dose modifications and concomitant antihypertensive treatment can effectively reduce TE-HTB severity

In the SELECT trial, 68% of the patients in the lenvatinib group received concomitant antihypertensive medication; 13% of the patients had dose reduction, 13% needed a dose interruption, and a discontinuation was necessary for 1% due to TE-HTN. This shows, that for the majority of the patients, antihypertensive therapy was an adequate means to control this adverse event [17].

No analyses regarding a possible correlation of hypertension occurrence/severity with study outcome were performed by the investigators of COSMIC-311 and DECISION. The COSMIC-311 trials were very recent, and further in-depth analyses might still follow, as the estimated study completion date is December 2022. In the DECISION trial, several parameters have been analyzed for their potential use as biomarkers for sorafenib efficacy, such as BRAF and RAS mutations, as well as serum thyroglobulin concentrations, sex, ethnic origin, age, DTC histology, Eastern Cooperative Oncology Group performance status, etc. [56]. It was found that tumor histology and burden, as well as sites of metastases, were independent predictive factors for a better PFS from sorafenib. However, the authors explicitly excluded AEs, as they argued that this exploratory analysis would incorporate too many uncertainties [86]. Only the SELECT trial has been analyzed with regards to an association of TE-HTN and outcome measures. In separate univariate analyses, TE-HTN was statistically significantly associated with both PFS (*p* < 0.01) and OS (*p* < 0.01), and in multivariate analyses it was only associated with OS (*p* = 0.04). In addition, there were hints that patients with TE-HTN also had an increased risk of developing congestive heart failure; however, this was not dependent on either TE-TN severity or duration of lenvatinib administration [17]. Table 5 gives a short summary of these findings.

### 3.3. Management of MKI-Induced Hypertension

Similar to essential hypertension, drug-induced hypertension needs to be well managed [17], both to reduce CV risks and to ensure the best treatment outcome possible, as the management of TEAEs allows the patient to continue the MKI treatment for a longer period of time [87]. In NCT01321554, there was a pre-specified plan for the management of TE-HTN, and BP was measured carefully both before and during the study (Table 6) [17,61].

During the study, patients with grade 2 hypertension or higher received antihypertensive medication. This treatment was administered to 68% of the patients treated with lenvatinib and 9% receiving placebo. Patients with a BP that was persistently grade 3 for more than three months, despite attempts of antihypertensive drug therapy, received treatment dose reductions or even interruptions of treatment [17]. When treating patients with antihypertensive drugs, the dose should be adapted according to the patients’ response and the tolerability of the antihypertensive treatment’s side effects [69]. The choice for the antihypertensive drug and possible lifestyle changes depends on a number of factors, such as the current understanding of MKI-induced hypertension pathophysiology, cardiovascular risk factors and cardiac comorbidities, left ventricular function, renal function, etc. [72].

The impact of dose interruption was also investigated in the study NCT01321554, as interruptions may potentially lead to a further disease progression. All patients in the treatment group experienced at least one dose interruption. For 134 patients, the interruption amounted to <10% of the total treatment duration, and for 127 patients to >10%. The study found that lenvatinib improved the outcome in RR-DTC in comparison to placebo, regardless of the duration of dose interruption. However, shorter durations of interruption showed better results. This is why the continuous evaluation of patients is necessary, so that TEAEs, such as hypertension, can be managed at an early stage and interruptions can be kept to a minimum [61].

The DECISISON trial began increased BP monitoring when grade 1 TE-HTN occurred. Upon asymptomatic grade 2 hypertension and DBP < 110 mmHg, antihypertensive therapy was started while sorafenib was continued. When patients suffered from grade 2 symptomatic/persistent hypertension or grade 3 hypertension or DBP ≥ 100 mmHg, antihypertensive therapy was continued and sorafenib was delayed until SBP ≤ 100 mmHg. Then, sorafenib was restarted with a dose reduction by one level. In the case of an uncontrolled SBP on therapy, the sorafenib dose was reduced by another level. Patients requiring delays of more than 14 days or more than two dose reductions, or who were suffering from grade 4 hypertension, had to discontinue the treatment [56].

## 4. Discussion

MKI-induced hypertension has the following causes: (1) reduced production of vasodilatory nitric oxide, (2) reduced prostacyclin production and (3) elevated production of vasoconstrictive endothelin-1 [88]. The underlying mechanisms of hypertension development were extensively reviewed in [76]. The MKI-induced increase in blood pressure is dependent on the application dose and has been suggested as a biomarker of treatment effectiveness [25,89]. Moreover, one might expect that these mechanisms of action of the MKIs induce a dose-dependent rise in blood pressure in nearly all patients after the first day or the first week of treatment [72].

A permanent control of baseline BP is necessary in order to avoid cardiovascular (CV) problems after MKI application [72]. Blood pressure should be monitored daily in patients with pre-existing hypertension. It is important that the BP is <120/80 mmHg before starting the MKI treatment [90]. Every week for the first two months and before the MKI infusion or cycle, the measuring of BP should be performed. In case of prehypertension (120 < SBP < 140 mmHg and 80 < DBP < 90 mmHg), other CV risk factors must be searched. When no other risk factors are detected, the application of MKI can begin, but under strict BP control [18].

Patients with an elevated systolic blood pressure of 135 mmHg to <160 mmHg or a diastolic blood pressure of 85 mmHg to <100 mmHg can still take MKIs, but an antihypertensive therapy has to be initiated. For patients who still have elevated blood pressure, a treatment break is necessary. Afterwards, in the case of lenvatinib treatment, the drug can be reinitiated at a lower dose when a stabilization of the blood pressure is achieved for at least 48 h [72].

It is important that the patients maintain an active lifestyle. In case of the detection of CV risk factors, pharmacological therapy with, for instance, calcium channel blockers (CCBs, such as amlodipine) is recommended. Antihypertensive drugs should be applied 3–7 days before the MKI therapy starts [18].

Recommendations for the antihypertensive therapy for patients with MKI-treated hypertension have been published in [18,72]. The recommended drugs are CCBs, angiotensin-converting enzyme inhibitors (ACEI, e.g., ramipril, lisinopril), angiotensin II receptor blockers (ARB, e.g., losartan, valsartan), diuretics (e.g., hydrochlorothiazide), β-adrenoceptor antagonists (BAA: e.g., nebivolol), nitrate derivates and endothelin receptor antagonists (ERA).

Endothelin receptor antagonists may be effective because the ET-1 level increases in MKI-treated cancer patients. The combination of two or three antihypertensive drugs may be effective in patients with MKI-induced hypertension [75,91].

Taken together, until today, there has been no clear guideline for treating MKI-induced hypertension, because patients have different anamnesis, constitutions and health statuses.

Early MKI-induced hypertension and the fast normalization of BP upon drug withdrawal suggest actions of the drugs on the microvasculature [92]. Therefore, the adverse effect of hypertension may serve as a biomarker of treatment efficacy [89].

Even though MKI treatment has shown good prognostic outcomes, the initiation of MKI treatment should be carefully considered, as this can lead to a range of different AEs of different grades, for example hypertension, and the treatment costs are high. To obtain the best benefit from the treatment, it is important to assess which patients should be treated and when the optimal time to start therapy is [38,93,94]. MKI treatment is systemic, and usually performed for longer periods of time. Therefore, this form of treatment should only be used in patients that definitely require this therapy, i.e., when other therapies are ineffective and disease progression is significant [93,95]. Yet postponing the initiation of treatment could possibly be associated with a poor outcome, as the efficiency is higher if the patients are still in a generally good condition [96,97]. To ensure that treatment is started at the optimal time, the surveillance of several parameters is necessary to follow disease progression [98].

Some factors may be prognostic for the efficacy and sensitivity of lenvativib treatment, e.g., baseline angiopoetin-2 (Ang2) levels may be predictive of the PFS in lenvatinib-treated patients, and BRAF mutations could be a negative prognostic factor in placebo patients with PTC [37]. The tumor doubling time (TDT) has been demonstrated to be prognostic, as, for example, a shorter doubling time could be correlated with increased treatment response. Other factors can be prognostic as well [62,99], e.g., localization of lesions [100], neutrophile-to-lymphocyte ratio (NLR) [3,101,102], and tumor size reduction within the first 8 weeks [62]. However, these results still need further research. Cabanillas et al. [31] showed that thyroglobin (Tg) response was correlated with the treatment response in some patients treated with cabozantinib, but further research regarding prognostic factors for Cabozantinib is still needed [31]. Reduced Tg has also been connected to improved PFS during therapy with lenvatinib [102,103].

The study NCT01321554 demonstrated that the differences in benefits in the group treated with lenvatinib compared to the placebo group were significantly higher in the elderly, who achieve improvements in both PFS and OS, although a higher toxicity was seen. A reason for this finding may be that the burden of the disease increases with age, or that older patients exhibit more co-morbidities [104].

It has to be taken into account that results seen in clinical trials with inclusion/exclusion criteria sometimes differ from the results in real-life (non-trial) settings. In the Dutch real-life study [34], the PFS in the treatment group was 9.7 months, compared with 18.3 months in the SELECT trial, and the toxicity was higher in the non-trial group. Although the difference could be explained by the different baseline characteristics [34], it is still important, as the results from the SELECT trial served as the basis for the approval of the lenvatinib treatment of RR-DTC [28,105], and the patients treated in the clinical practice might not fit the inclusion/exclusion criteria [38]. The same tendency was seen in a Korean retrospective study [100] and in Locati et al.’s study [48]. Different studies have found varying results when comparing real-life and clinical trials, further indicating the challenge of drug approval and usage, as not all patients derive a similar good outcome from MKI treatment as was seen in clinical trials, and some patients even develop resistance to the therapy. This should affect the decision as to who will receive MKI treatment, especially when taking TEAEs into account, as this could negatively affect the patients’ quality of life. However, many of the studies showed that lenvatinib has a high effectiveness and an acceptable safety profile in a real-life setting [32,35,39,81,97,99,100,106,107,108,109]. In a retrospective analysis published by Gianoukakis et al. [110], it was shown that lenvatinib responders in the SELECT trial had an increased PFS and duration of response compared with the primary analysis, indicating the importance of the assessment of possible responses before the treatment is initiated [110].

The results of clinical trials investigating the efficacy and safety of MKIs demonstrate that patients with grade 2 and 3 hypertension achieved a better survival rate than patients with normal blood pressure [89,111]. The development of hypertension correlated with a longer PFS and OS. The phase 3 SELECT trial (NCT01321554) was a global, randomized, double-blind, multicenter clinical trial, and it investigated 392 patients with thyroid cancer. It was found that the PFS values in the groups treated with lenvatinib with (*n* = 190) or without (*n* = 71) drug-induced hypertension were 18.8 months (95% CI, 16.5 months to NE) and 12.9 months (95% CI, 7.4 months to NE), respectively, HR 0.59 (95% CI 0.39 to 0.88, log-rank *p* = 0.0085). The grade of hypertension was not found to matter. It should be noted that the authors mention an analytic complication due to the high proportion of hypertensive patients at baseline [17]. At baseline, 56% of the patients receiving lenvatinib and 57% of the patients with placebo suffered from hypertension. More patients randomized to lenvatinib experienced treatment-emergent HTN (73%) as compared to the placebo group (15%) [17]. Grade ≥ 3 hypertension was measured in 116 (44%, lenvatinib group) and 5 patients (4%, placebo group). Patients with RR-DTC treated with lenvatinib showed a significant improvement in PFS and a high ORR compared to patients who received placebo [58]. Drug-induced hypertension in the lenvatinib group was significantly associated with both PFS and OS in the univariate analysis; in the multivariate analysis, only OS reached significance [17]. This correlation is also seen in the Dutch real-life study [34] in a post hoc analysis, where the median PFS was 16.6 months (95% CI: 11.1 to 22.0) in the hypertension group vs. 5.1 (95% CI: 3.8 to 6.5) in the non-hypertensive group [34].

The discovery and management of TEAEs at an early stage is important to achieve the best treatment benefit. In addition, this is relevant to compliance, drug continuation, and the need for dose reductions [71,94,112,113]. Jasim et al. [113] found that the AEs occurred early after the initiation of lenvatinib treatment, within months, but generally the tolerability was good, especially compared to other common treatments used for cancer [113]. The retrospective study published by Suyama et al. [114] showed that hypertension was the main reason for the dose reduction within the first 10 days [114], and Haddad et al. measured that the median time to onset of the first any-grade TEAE was 12 weeks [115]. According to Porcelli et al. [116], most TEAEs occur within 12 months of treatment initiation, and rarely at a later treatment stage, further stressing the importance of early TEAE management and drug continuation in the early stages of treatment [116].

The treatment of drug-induced hypertension consists of the same drug-groups as the therapy of essential hypertension, or in the case of resistant hypertension, dose reduction/interruption may be necessary [15,87]. Some problems may occur, because some of the drug’s function by promoting VEGF secretion, and it is not fully understood if this affects the function of MKI treatment. The recommended antihypertensive drugs, according to Sueta et al., are ACEI and ARBs, which improve the dysfunction of the vascular endothelium and lead to NO release [71]. Suyama et al. [114] suggested that ACEI and CCB could be useful in the treatment of hypertension and in the protection of the vascular endothelial cells [114]. CCBs could pose an issue, as these drugs are metabolized by the same enzymes as MKIs, which can negatively affect the treatment outcome [117]. There is still a need for further research to determine the best antihypertensive drugs to treat MKI-induced hypertension [71,82].

The outcome in patients treated with lenvatinib compared to placebo is improved, regardless of the duration of interruption, which is promising for the patients. It should be noted that some studies discuss the flare phenomena, i.e., the short acceleration of tumor progression, over as little as a few days, after the interruption of TKI treatment. No interruption of treatment would be ideal. Tumor-related symptoms can reduce the treatment efficiency [33,38,105,118]. The clinical trial NCT02702388 and Yamazaki et al. [119] investigated whether a lower starting dose could have the same treatment benefits without the treatment-related toxicities, which could lead to fewer dose interruptions and thereby a better treatment outcome. De Leo et al. [96] further suggested that an individualized starting dose could improve the treatment result, as the first months of treatment are of utmost importance, and interruption of the treatment at this time should be avoided if possible [96]. The aim should be to obtain the highest dose possible with the lowest rate of TEAEs in order to maximize efficiency [120]. Masaki et al. and Robinson et al. demonstrated that lenvatinib induces a rapid early treatment response and immediately shrinks the tumor size within 8 weeks. Thereafter, the tumor size is further decreasing, but at a slower pace, thus the early treatment phase is critical [62,121]. The risk of disease progression, “flare up”, when treatment is interrupted means that it is important to have subsequent treatment possibilities that can be initiated without hesitation [118].

Cabozantinib has just been approved as a salvage treatment for RR-DTC, and is indicated when other MKI treatments, such as lenvatinib, fail. The circumstances leading to further treatment response when using cabozantinib as a second-line treatment are not completely understood, but they could be related to c-MET inhibition, as previous VEGFR inhibition induces c-MET-driven resistance [31,122], as VEGFR inhibition can lead to hypoxia, which may lead to enhancements in c-MET expression, thus enhancing tumor invasion [29,70]. The risk of resistance development and tumor escape mechanisms means that there is still a need for further developments in other multi-targeted therapies to improve cancer therapy [19].

Recently, Zheng et al. [123] published the results of a randomized, phase III study of lenvatinib in Chinese patients with RR-DTC. The authors showed that the PFS was significantly longer in patients treated with lenvatinib versus placebo, similar to the results from SELECT. In total, 103 patients received lenvatinib and 48 patients were enrolled in the placebo group.

The PFS was significantly longer with lenvatinib treatment (*n* = 103; median 23.9 months; 95% confidence interval (CI) 12.9—not estimable) versus placebo (*n* = 48; median 3.7 months; 95% CI 1.9–5.6; hazard ratio = 0.16, 95% CI 0.10–0.26; *p* < 0.0001). The OR was 69.9% (95% CI 61.0–78.8) in lenvatinib patients and 0% (95% CI 0–0) in the placebo group. Treatment-emergent AEs led to lenvatinib discontinuation in 8.7% of patients. Overall, MKI-induced AEs of grade ≥3 occurred in 87.4% of patients in the lenvatinib arm, the most common being hypertension (lenvatinib: 64 patients, 62.1% vs. placebo: 4 patients, 8.3%) [123]. The randomization phase consisted of the study–treatment cycles, during which patients received a starting dose of 24 mg/day lenvatinib or placebo p.o. In case a grade 2 or grade 3 lenvatinib-related AE was detected, the lenvatinib treatment was interrupted until the AE was tolerable and reached grade ≤1 or baseline. Lenvatinib was then applied at a reduced dose, with a reduced scheme of 20 mg, 14 mg, and then 10 mg/day for each successive toxicity occurrence [123].

Kim et al. [124] retrospectively reviewed the medical records of patients with advanced or metastatic DTC treated for ≥6 months with lenvatinib or sorafenib in South Korea. A total of 71 medical records (lenvatinib, *n* = 23; sorafenib, *n* = 48) were reviewed. One of the most common AEs of any grade in the lenvatinib group was hypertension (78.3%). In the sorafenib group, hypertension was detected at 43.8%. The incidence of hypertension of any grade was significantly higher (*p* = 0.006) in patients treated with lenvatinib compared with those treated with sorafenib. The incidence of grade ≥3 hypertension in patients treated with lenvatinib was *n* = 17 (73.9%) vs. those treated with sorafenib *n* = 15 (31.3) (*p* < 0.001) [124].

In general, lenvatinib compared to sorafenib or cabozantinib is associated with a higher incidence of severe hypertension, which possibly indicates a more potent inhibitory effect [67].

The COSMIC-311 trial (NCT03690388) is a randomized, double-blind, placebo-controlled phase 3 trial investigating cabozantinib in patients with RR-DTC [54] who had been earlier treated with either sorafenib or lenvatinib, or both. In total, 187 patients were enrolled in the study and randomly assigned to cabozantinib (*n* = 125) or placebo (*n* = 62). Cabozantinib significantly prolonged the PFS in these patients. Grade 3 hypertension occurred in 10 (8%) and grade 4 hypertension in 1 (1%) of the cabozantinib group vs. 2 (3%) and 0 patients in the placebo group [54].

Taken together, patients who will receive lenvatinib or sorafenib medications’ blood pressure measurements should be performed daily in cases of pre-existing hypertension. Otherwise, blood pressure measurements are necessary from 1 week after the initiation of lenvatinib, and weekly for the first 2 months. Patients with diagnosed hypertension of ≥135 mmHg to <160 mmHg or diastolic blood pressure ≥85 mmHg to <100 mmHg should receive lenvatinib, but antihypertensive therapy should be initiated or intensified. For patients who remain hypertensive, a treatment break can be considered with lenvatinib reinitiated at a reduced dose once the patient’s blood pressure has stabilized for at least 48 h. Table 7 summarizes the dose management applied in clinical trials for MKI treatment in thyroid cancer patients.

Prophylaxis, a healthy life style, and regular BP measurements with appropriate short treatment breaks and, for persistent adverse events, dose reductions, are recommended to enable patients to remain on the optimal dose regimen [56,72,125].

## 5. Conclusions

Treatments with lenvatinib, sorafenib and cabozantinib show promising results and an improved PFS in RR-DTC. Unfortunately, AEs are a part of most treatments, including MKIs, but for most patients treated with MKIs, the toxicities are well-tolerated and relatively easy to manage, making this therapy a good option for most patients. When treating with MKIs, it is important to assess how the benefit of treatment and the TEAEs affect the patients’ quality of life, and if the treatment should be continued. The specific mechanisms regarding both treatment efficiency and the development of TEAEs needs further investigation to offer patients the best treatment regime and prognosis possible. So far, in the clinical context, lenvatinib is the widely preferred first-line drug, based on its superior PFS benefit, improved OS in patients over 65 years, higher objective response rate, and an overall more tolerable and manageable spectrum of side effects compared to sorafenib [127]. The much higher incidence and the severity of TE-HTN induced by lenvatinib play only minor roles in these considerations, as they are outweighed by other, less well-manageable AEs.

## Figures and Tables

**Table 1:** Characteristics of sorafenib, lenvatinib, and cabozantinib.

Drug	Target	Half-Life in Plasma	Metabolism	Approval for DTC	Structure
Sorafenib	VEGFR-2 and -3 PDGFR, c-Kit, RET/PTC, RAF [41]	36 h [41]	Hepatic CYP3A4 and UGT1A9 [42]	2013 [43]	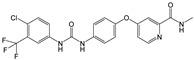
Lenvatinib	VEGFR 1-3, PDGFR-α, RET, c-KIT, FGFR 1-4 [3]	28 h [44]	Hepatic CYP3A4 [45]	2015 [28]	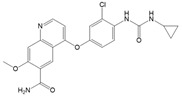
Cabozantinib	VEGFR-2, c-MET, RET [31]	100–120 h [46]	Hepatic CYP3A4 [46]	2021 [47]	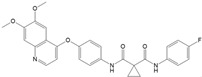

Structures were drawn with ChemDraw Professional 17 (Perkin Elmer Informatics, Waltham, MA, USA).

**Table 2:** Overview of the three pivotal phase 3 trials for cabozantinib, sorafenib, and lenvatinib.

Title	Design	Number of Participants	Doses and Cycles	Results	Status
A Study of Cabozantinib Compared with Placebo in Subjects with Radioiodine-refractory Differentiated Thyroid Cancer Who Have Progressed After Prior VEGFR-targeted Therapy (COSMIC-311 Trial) NCT03690388 [54]	Interventional, randomized, double blind	Planned 300 Enrolled187	60 mg or 20 mg cabozantinib or placebo equivalent once daily	PFS vs. placebo: median not reached (96% CI 5.7–not estimable) versus 1.9 months (1.8–3.6); hazard ratio 0.22 (96% CI 0.13–0.36; *p* < 0.0001)	Active, not recruiting
A Double-Blind Randomized Phase III Study Evaluating the Efficacy and Safety of Sorafenib Compared to Placebo in Locally Advanced/Metastatic RAI-Refractory Differentiated Thyroid Cancer (DECISISON) NCT0098428 [55,56,57]	Interventional, double blind, randomized	417	Sorafenib 800 mg/day (400 mg every 12 h) Placebo twice daily (approximately every 12 h).	PFS vs. placebo: hazard ratio, 0.59 (95% CI 0.45–0.76); *p* < 0.0001; median 10.8 vs. 5.8 months	Completed
A Multicenter, Randomized, Double-Blind, Placebo-Controlled Trial of Lenvatinib (E7080) in 131I-Refractory Differentiated Thyroid Cancer (SELECT) NCT01321554 [37,58,59,60,61,62,63]	Interventional, double blind, randomized	392	Randomization phase: Starting dose—24 mg Lenvatinib orally (two 10 mg tablets and one 4 mg tablet) once daily, continuously, or placebo matching the lenvatinib treatment.	PFS Treatment: 18.3 months Placebo: 3.6 months HR: 0.21 (99% CI: 0.14 to 0.31) ORR Treatment: 64.8% Placebo: 1.5%.	Completed

**Table 3:** Definition of hypertension and classification of BP.

Condition	Systolic Blood Pressure (mmHg)		Diastolic Blood Pressure (mmHg)
Optimal	<120	and	<80
Normal	120–129	and/or	80–84
High normal	130–139	and/or	85–89
Grade 1 hypertension	140–159	and/or	90–99
Grade 2 hypertension	160–179	and/or	100–109
Grade 3 hypertension	≥180	and/or	≥110
Isolated systolic hypertension	≥140	and	<90

Adapted from the European Society of Hypertension and European Society of Cardiology guidelines 2018 [78].

**Table 4:** Overview of top 5 adverse effects found in clinical trials of lenvatinib, cabozantinib, and sorafenib in RR-DTC.

Title	Daily Dose	Most Frequent AEs (Not Including Serious)	Most Frequent Serious AEs
Evaluating the Safety and Efficacy of Oral Lenvatinib in Medullary and Iodine-131 Refractory, Unresectable Differentiated Thyroid Cancers, Stratified by Histology NCT00784303 [83]	24 mg lenvatinib	Hypertension 77.59% Diarrhea 68.97% Fatigue 60.34% Decreased appetite 55.17% Nausea 51.72%	Hypotension 6.9% Dehydration 6.9% Hypertension 3.45% Renal failure 3.45 Pneumonia 3.45%
A Trial of Lenvatinib (E7080) in Subjects with Iodine-131 Refractory Differentiated Thyroid Cancer to Evaluate Whether an Oral Starting Dose of 18 Milligram (mg) Daily Will Provide Comparable Efficacy to a 24 mg Starting Dose, But Have a Better Safety Profile NCT02702388	24 mg or 18 mg lenvatinib (starting dose)	24 mg: Hypertension 57.33% Diarrhea 56% Proteinuria 42.67% Nausea 40.00% Weight loss 36.00% 18 mg: Hypertension 51.95% Diarrhea 51.95% Weight loss 42.86% Stomatitis 38.57% Nausea 35.06%	24 mg: Total 33.33% Malignant neoplasm progression 4% Pneumothorax 2.67% Hypertension 1.33% 18 mg: Total 40.26% Osteoarthritis 2.60% Pathological fracture 2.60% Malignant neoplasm progression 2.60% Malignant pleural effusion 2.60%
A Multicenter, Randomized, Double-Blind, Placebo-Controlled, Trial of Lenvatinib (E7080) in 131I-Refractory Differentiated Thyroid Cancer (DTC) (SELECT) NCT01321554 [58]	24 mg lenvatinib Placebo matching the lenvatinib treatment.	Hypertension 69.35% Diarrhea 69.73% Loss of appetite 56.70% Weight loss 54.41% Nausea 48.66%	Pneumonia 4.6% Hypertension 3.83 Dehydration 3.45% General physical health deterioration 2.68% Pulmonary embolism 2.3%
A Study of E7080 in Subjects with Advanced Thyroid Cancer NCT01728623	24 mg lenvatinib	Hypertension 90.20% Palmar-plantar erythrodysaesthesia syndrome 76.47% Loss of appetite 76.47% Fatigue 72.55% Proteinuria 60.78%	Decreased appetite 13.73% Malignant neoplasm progression 7.84% Pneumonia 5.88% Nausea 3.92% Laryngeal stenosis 3.92%
Cabozantinib-S-Malate in Treating Patients with Refractory Thyroid Cancer NCT01811212 [31]	60 mg cabozantinib S-malate	Liver transaminase elevation 80% Palmar-plantar erythrodysesthesia 76% Fatigue 76% Diarrhea 72% Nausea 64% … Hypertension 48%	Left ventricular systolic dysfunction 4% Osteonecrosis of the jaw 4% Asymptomatic increased lipase 4% Meningitis 4% Pneumonia 4%
A Study of Cabozantinib Compared with Placebo in Subjects with Radioiodine-refractory Differentiated Thyroid Cancer Who Have Progressed After Prior VEGFR-targeted Therapy (COSMIC-311 Trial) NCT03690388 [54]	60 mg or 20 mg cabozantinib or placebo equivalent once daily	Diarrhea 44% Palmar-plantar erythrodysesthesia 35% Alanine aminotransferase increased 23% Aspartate aminotransferase increased 23% Nausea 21% … Hypertension 19%	Palmar-plantar erythrodysesthesia 10% Hypertension 9% Fatigue 8% Diarrhoea 7% Hypocalcaemia 7%
Safety and Efficacy of Sorafenib in Patients with Advanced Thyroid Cancer: a Phase II Clinical Study NCT02084732 [84]	800 mg/day sorafenib	Hypertension 42.1% Hand/food Erythema 36.8% Diarrhea 31.5% Muscle pain 21% Rash 21%	Hypertension 56,8% Hand/food Erythema 31.5% Diarrhea 26.2% Rash 15.7% Acute myocardial infarction 5.2%
Sorafenib as Adjuvant to Radioiodine Therapy in Non-Medullary Thyroid Carcinoma NCT00887107 [85]	800 mg/day sorafenib	Weight loss 47.6% Diarrhea 50% Alopecia 47% Rash 47% Hand foot syndrome 43.6% … Hypertension 25.4%	Hand foot syndrome 21.8% Hypertension 15% Weight loss 8.4% Myocardial infarction 3% Congestive heart disease 3%
A Double-Blind Randomized Phase III Study Evaluating the Efficacy and Safety of Sorafenib Compared to Placebo in Locally Advanced/Metastatic RAI-Refractory Differentiated Thyroid Cancer (DECISISON) NCT00984282 [56]	800 mg/day sorafenib	Alopecia 67.1% Diarrhea 62.8% Hand–foot skin reaction 56% Rash 45.4% Fatigue 44% Hypertension 30.9%	Hand–foot skin reaction 20.3% Hypertension 9.7% Hypocalcemia 9.2 Diarrhea 5.8% Fatigue 5.8%

If hypertension was not among the top 5 AEs, it is still listed, separated by “…” from the rest.

**Table 5:** Occurrence of TE-HTN and TE-HTN dose modifications.

Drug	TE-HTN Grades 1–2 (%)	TE-HTN ≥ Grade 3 (%)	Dose Reductions (%)	Dose Interruptions (%)	Discontinuations (%)	TE-HTN-Related Deaths (%)
Lenvatinib [17,58]	26	41.8	13	13	1	0
Sorafenib [55,56]	30.9	9.7	5.8	7.7	0.5	0
Cabozantinib [54]	19	9	<7	n/a	0.8	0

All data derived from COSMIC-311, SELECT, and DECISION trials. n/a: not available.

**Table 6:** Pre-specified treatment plan in NCT01321554.

BP Level (mmHg)	Grade of AE	Half-life in plasma
SBP < 140, DBP < 90	Grade 1 AE	No treatment needed
SBP 140–159, DBP 90–99	Grade 2 AE	Need for antihypertensive medication
SBP ≥ 160, DBP ≥ 100	Grade 3 AE	Need for antihypertensive medication If persistent for 3 consecutive months, dose reductions are required
Life-threatening	Grade 4 AE	Urgent intervention is indicated, possibly treatment interruption

Management plan for drug-induced hypertension at different grades [17,61,77].

**Table 7:** Hypertension management in clinical trials. Dose modifications for MKIs.

Drug	Clinical Trial	Dose Level	Daily Dose	References
Lenvatinib (Lenvima^®^)	SELECT (NCT01321554)	Recommended daily dose	24 mg p.o. once daily: 2 × 10 mg and 1 × 4 mg in capsules	[44,58,72]
		Dose reduction No. 1	20 mg p.o. once daily: 2 × 10 mg in capsules	
		Dose reduction No. 2	14 mg orally once daily (1 × 10 mg capsule plus 1 × 4 mg capsule)	
		Dose reduction No. 3	10 mg p.o. once daily: 1 × 10 mg capsule	
Lenvatinib (Lenvima^®^)	Lenvatinib in Chinese patients with RR-DTC	Starting dose	24 mg/day p.o.	[123]
		In case of intolerable grade 2 or grade 3 HTN; lenvatinib treatment interruption until the toxicity had resolved to grade ≤1 or baseline.		
		Lenvatinib treatment was then resumed at a reduced dose:		
		Dose reduction No. 1	20 mg/day	
		Dose reduction No. 2	14 mg/day	
		Dose reduction No. 3	10 mg/day	
Lenvatinib (Lenvima^®^) Sorafenib (NEXAVAR^®^)	Lenvatinib and Sorafenib in South Korean patients with advanced and metastatic RR-DTC	Lenvatinib arm: starting dose	20 mg/day capsules	[124]
		Sorafenib arm: starting dose	800 mg/day tablets	
	
		Final lenvatinib doses:		
		*N* = 7 (30.4%)	10 mg daily	
		*N* = 2 (8.7%)	14 mg daily	
		*N* = 14 (60.9%)	20 mg daily	
		Dose reduction for AEs:		
		*N* = 8 (34.8%)		
		Discontinuation of lenvatinib:		
		AEs		
		*N* = 1 (4.3%)		
		Disease progression:		
		*N* = 0		
		Death:		
		*N* = 2 (8.7%) Financial issues:		
		*N* = 1 (4.3%)		
	
		Initial sorafenib dose:		
		*N* = 12 (25.0)	≤400 mg dail	
		*N* = 16 (33.3)	600 mg daily	
		*N* = 20 (41.7)	800 mg daily	
		Final sorafenib dose:		
		*N* = 20 (41.7)	≤400 mg daily	
		*N* = 21 (43.8)	600 mg daily	
		*N* = 7 (14.6)	800 mg daily	
		Dose reduction for AEs:		
		*N* = 27 (56.3)		
		Discontinuation of sorafenib:		
		AE		
		*N* = 4 (8.3)		
		Disease progression:		
		*N* = 17 (35.4)		
		Death:		
		*N* = 5 (10.4)		
Sorafenib (NEXAVAR^®^)	DECISION (NCT00984282)	Recommended daily dose	2 × 400 mg p.o.	[56]
		Study drug dose interruption or sequential reduction No. 1	600 mg (divided doses: 400 and 200) p.o. daily	
		Dose reduction No. 2	400 mg (divided 2 × 200) p.o. daily	
		Dose reduction No. 3	200 mg daily	
Cabozantinib	COSMIC-311 (NCT03690388)	Cabozantinib		[54]
		AEs managed by dose modifications:	60 mg/day tablets p.o.	
		The median daily dose was 42.0 mg (IQR 32.2–54.5) with cabozantinib and 60.0 mg (52.9–60.0) with placebo.	Reductions from 60 mg to 40 mg and then to 20 mg daily	
Cabozantinib	EXAM (NCT00704730) MTC (medullary thyroid carcinoma) patients	Cabozantinib starting dose:	140 mg/day	[125,126]
		AEs managed with concomitant medications, dose interruptions, and dose reductions;		
		SAEs were more frequent in cabozantinib- versus placebo-treated patients. For hypertension: 2.3% (5 of 214 cabozantinib) vs. 0% (0 of 109 placebo).		

## Data Availability

Not applicable.

## Abbreviations

AE(s)	adverse effect(s)
ATC	anaplastic thyroid cancer
BP	blood pressure
CV	cardiovascular
DBP	diastolic blood pressure
DTC	differentiated thyroid cancer
EGF	epidermal growth factor
EGFR	epidermal growth factor receptor
FGFR	fibroblast growth factor receptor
FTC	follicular thyroid cancer
HCC	hepatocellular carcinoma
HTC	Hürthle cell thyroid cancer
HGF	hepatocyte growth factor
HGFR	hepatocyte growth factor receptor
MTC	medullary thyroid cancer
MKI	multikinase inhibitor
ORR	overall response rate
OS	overall survival
PTC	papillary thyroid cancer
PDGF	platelet-derived growth factor
PDGFR	platelet-derived growth factor receptor
PDTC	poorly differentiated thyroid cancer
PFS	progression-free survival
RAI	radioactive iodine
RCC	Renal clear cell carcinoma
RR-DTC	RAI refractory DTC
RTK	receptor tyrosine kinases
SBP	systolic blood pressure
TEAE	treatment-emerged adverse effect
TE-HTN	treatment-emerged hypertension
TC	thyroid cancer
TK(s)	tyrosine kinase(s)
VEGFR	vascular endothelial growth factor receptor
VEGF	vascular endothelial growth factor
